# Transcranial Doppler as a screening test to exclude intracranial hypertension in brain-injured patients: the IMPRESSIT-2 prospective multicenter international study

**DOI:** 10.1186/s13054-022-03978-2

**Published:** 2022-04-15

**Authors:** Frank A. Rasulo, Stefano Calza, Chiara Robba, Fabio Silvio Taccone, Daniele G. Biasucci, Rafael Badenes, Simone Piva, Davide Savo, Giuseppe Citerio, Jamil R. Dibu, Francesco Curto, Martina Merciadri, Paolo Gritti, Paola Fassini, Soojin Park, Massimo Lamperti, Pierre Bouzat, Paolo Malacarne, Arturo Chieregato, Rita Bertuetti, Raffaele Aspide, Alfredo Cantoni, Victoria McCredie, Lucrezia Guadrini, Nicola Latronico

**Affiliations:** 1 Department of Anesthesiology, Intensive Care and Emergency, Spedali Civili University Hospital, 25123 Brescia, Italy; 2 Department of Surgical Specialties, Radiological Sciences and Public Health, University of Brescia, Brescia, Italy; 3 Unit of Biostatistics and Bioinformatics, Department of Molecular and Translational Medicine, University of Brescia, Brescia, Italy; 4 Policlinico San Martino, Dipartimento di Scienze Chirurgiche ed Integrate, University of Genoa, Genoa, Italy; 5 Department of Intensive Care, Erasme Hospital, Universitè Libre de Bruxelles, Brussels, Belgium; 6 Neurosurgical Intensive Care, Fondazione Policlinico Universitario “A. Gemelli”, IRCCS, Rome, Italy; 7 Department of Anesthesiology and Intensive Care, Hospital Clínic Universitari, University of Valencia, Valencia, Spain; 8 Neurointensive Care Unit, San Gerardo Hospital, ASST-Monza, Monza, Italy; 9 University of Milano-Bicocca, Milan, Italy; 10 Cerebrovascular Center of Neurological Institute, Cleveland Clinic, Abu Dhabi, United Arab Emirates; 11 Department of Neurointensive Care, Grande Ospedale Metropolitano, Niguarda, Milan, Italy; 12 Department of Anesthesiology and Critical Care, Azienda Ospedaliera Universitaria Pisana, Pisa, Italy; 13 Department of Anesthesiology and Critical Care, Papa Giovanni XXIII Hospital, Bergamo, Italy; 14 Department of Anesthesia and Intensive Care, Legnano Hospital, Legnano, Italy; 15 Division of Critical Care Neurology, Columbia University Irving Medical Center, New York, USA; 16 Anesthesiology Institute, Cleveland Clinic, Abu Dhabi, United Arab Emirates; 17 Division of Anesthesiology and Intensive Care, Grenobles, France; 18 Anesthesia and Intensive Care Unit, Istituto delle Scienze Neurologiche di Bologna, Bologna, Italy; 19 Neurointensive Care, ASST-SETTELAGHI, Varese, Italy; 20 Toronto Western Hospital, University Health Network, Toronto, ON Canada

**Keywords:** Intracranial pressure, Noninvasive monitoring, Brain injury, Intracranial hypertension

## Abstract

**Background:**

Alternative noninvasive methods capable of excluding intracranial hypertension through use of transcranial Doppler (ICP*tcd*) in situations where invasive methods cannot be used or are not available would be useful during the management of acutely brain-injured patients. The objective of this study was to determine whether ICP*tcd* can be considered a reliable screening test compared to the reference standard method, invasive ICP monitoring (ICP*i*), in excluding the presence of intracranial hypertension.

**Methods:**

This was a prospective, international, multicenter, unblinded, diagnostic accuracy study comparing the index test (ICP*tcd*) with a reference standard (ICP*i*), defined as the best available method for establishing the presence or absence of the condition of interest (i.e., intracranial hypertension). Acute brain-injured patients pertaining to one of four categories: traumatic brain injury (TBI), subarachnoid hemorrhage (SAH), intracerebral hemorrhage (ICH) or ischemic stroke (IS) requiring ICP*i* monitoring, were enrolled in 16 international intensive care units. ICP*i* measurements (reference test) were compared to simultaneous ICP*tcd* measurements *(*index test) at three different timepoints: before, immediately after and 2 to 3 h following ICP*i* catheter insertion. Sensitivity, specificity, positive (PPV) and negative predictive values (NPV) were calculated at three different ICP*i* thresholds (> 20, > 22 and > 25 mmHg) to assess ICP*tcd* as a bedside real-practice screening method. A receiver operating characteristic (ROC) curve analysis with the area under the curve (AUC) was used to evaluate the discriminative accuracy and predictive capability of ICP*tcd.*

**Results:**

Two hundred and sixty-two patients were recruited for final analysis. Intracranial hypertension (> 22 mmHg) occurred in 87 patients (33.2%). The total number of paired comparisons between ICP*tcd* and ICP*i* was 687. The NPV was elevated (ICP > 20 mmHg = 91.3%, > 22 mmHg = 95.6%, > 25 mmHg = 98.6%), indicating high discriminant accuracy of ICP*tcd* in excluding intracranial hypertension. Concordance correlation between ICP*tcd* and ICP*i* was 33.3% (95% CI 25.6–40.5%), and Bland–Altman showed a mean bias of -3.3 mmHg. The optimal ICP*tcd* threshold for ruling out intracranial hypertension was 20.5 mmHg, corresponding to a sensitivity of 70% (95% CI 40.7–92.6%) and a specificity of 72% (95% CI 51.9–94.0%) with an AUC of 76% (95% CI 65.6–85.5%).

**Conclusions and relevance:**

ICP*tcd* has a high NPV in ruling out intracranial hypertension and may be useful to clinicians in situations where invasive methods cannot be used or not available.

*Trial registration*: NCT02322970.

**Supplementary Information:**

The online version contains supplementary material available at 10.1186/s13054-022-03978-2.

## Introduction

Intracranial hypertension is a common and severe complication of acute brain injury [1]. If diagnosis is delayed, intracranial hypertension can lead to brain herniation with compression of the brainstem and distortion of brain parenchyma and vessels, and ultimately death [1]. Both the presence of intracranial hypertension and its burden, defined as the total amount of time spent above different thresholds of intracranial pressure (ICP), have been correlated with poor outcome [2]. The reference standard for diagnosing intracranial hypertension is the invasive measurement of ICP using ventricular drains or cerebral catheters and microsensors placed through a burr hole drilled in the skull [3]. However, invasive ICP measurement may not be available in certain situations, such as in underserved rural areas, in developing countries with limited resources or in settings outside the intensive care unit (ICU) and neurosurgical departments [4]. In several conditions, such as liver failure, preeclampsia, encephalitis, meningitis and stroke, the role of invasive ICP monitoring is not well established or the risk–benefit ratio remains unclear [5–9]. Moreover, invasive insertion of intracranial transducers can result in important complications, including infection and hemorrhage [10, 11]. For these reasons, the presence of intracranial hypertension is often estimated based on noninvasive screening methods, including neurological examination, brain imaging or cerebral ultrasonography (i.e., optic nerve sheath diameter and transcranial Doppler, TCD) [12]. These screening tests are more easily accessible, less expensive and time-consuming, less invasive, and hence, less physically and psychologically discomforting for patients. However, the diagnostic accuracy of these methods remains poorly defined. A TCD-derived formula to estimate cerebral perfusion pressure (CPP*e*) and ICP (ICP*tcd*) has been available for more than 20 years [13, 14]. In 2017, a multicenter pilot study (IMPRESSIT: Invasive vs. noninvasive Measurement of intracranial PRESSure in brain Injury Trial) reported that the ICP*tcd* threshold of 24.3 mmHg was associated with a 100% sensitivity and 100% negative predictive value (NPV) in excluding the presence of intracranial hypertension (ICP > 20 mmHg) [15]. Based on these preliminary findings, and to definitively answer whether ICP*tcd* represents an accurate noninvasive ICP measurement method, we designed a prospective, international, multicenter study to evaluate the discriminant accuracy of TCD in excluding intracranial hypertension in a broad and representative cohort of acute brain-injured patients.

## Methods

### Study design

This prospective, international, multicenter, diagnostic accuracy study is reported according to the Standards for Reporting of Diagnostic Accuracy Studies (STARD) guidelines (Additional file 1: Appendix 1) [16] and involved comparing the index test, ICP*tcd*, with a reference standard (invasive ICP, ICPi), defined as the best available method for establishing the presence or absence of the condition of interest (i.e., intracranial hypertension). The study was unblinded, and the reference standard results were available to the performers of the index test.

*ICPtcd measurement* (*index test).* We used a method based on a mathematical model which comprises parameters derived from TCD flow velocities and arterial blood pressure, which is described in detail elsewhere [13–15]. Briefly, CPPe is first estimated as: MAP*FVd /FVm + 14, where MAP is the mean arterial blood pressure and FVd and FVm are, respectively, the MCA *diastolic* and *mean* blood flow velocities derived from the TCD measurements. ICP*tcd* is then calculated as: MAP–CPPe.

The insonation technique of the middle cerebral artery (MCA) was standardized across all sites: A low-frequency pulsed 2 MHz ultrasound probe was placed over the acoustic temporal window for insonation of the M1/M2 section of MCA at a depth ranging from 45 to 55 mm. The acoustic window ipsilateral to the side of the ICP*i* placement was preferred. Cerebral blood flow velocities were assessed by using either of two technologies, TCD or transcranial color-coded Doppler (TCCD). Regarding the various ultrasound machine companies, no limitations nor specifications were requested by the protocol since the ICP*tcd* method is based on a calculated formula derived from standard TCD parameters [13, 14].

**Fig. 1:**
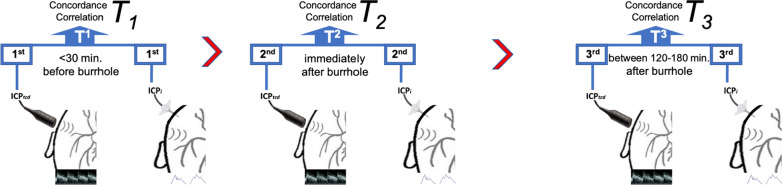
The three time frames (*T*_1_, *T*_2_, *T*_3_) of paired invasive (ICP*i*) and noninvasive (ICPtcd) measurements of intracranial pressure (ICP). At TIME 1 (*T*_1_), ICP*tcd* was obtained shortly before performing the burr hole procedure and was compared to the first ICP*i* reading once the invasive probe was positioned. At TIME 2 (*T*_2_) and TIME 3 (*T*_3_), paired ICPtcd and ICPi were obtained immediately after TIME 1 and 2 to 3 h following the second reading

For each patient enrolled into the study, a total of three comparisons of ICPi and ICPtcd were made in three different time frames, as described previously in the IMPRESSIT pilot study (Fig. 1).

At TIME 1, ICP*tcd* was obtained shortly before performing the burr hole procedure and was compared to the first ICP*i* reading once the invasive probe was positioned. An effort was made to limit the cerebrospinal fluid leakage during intraventricular catheter placement which may cause changes in ICP. At TIME 2 and TIME 3, paired ICP*tcd* and ICP*i* were obtained immediately after TIME 1 and 2 to 3 h following the second reading. This later time lapse was chosen for two reasons: First, it was deemed necessary to permit any variations of cerebral hemodynamics induced by the ICP*i* device insertion maneuver to stabilize to a steady state; second, once the invasive device is inserted, a specific physiologically based time lapse becomes less relevant since any further variations in cerebral hemodynamics would automatically influence both the invasive and noninvasive measurement.

#### ICPi measurement (reference test)

The decision to insert invasive ICP monitoring was independent from the study protocol and was made by the physician in charge of the patient based on the international guidelines for TBI patients, and neurological signs (i.e., altered pupillary diameter and light reaction, altered reflex motor response), clinical course (i.e., rapid neurological worsening) and neuroradiological findings (midline shift, cerebral ventricles dilatation or compression, signs of transtentorial brain herniation, obliteration of the brainstem cisterns) for SAH, ICH and IS patients. Arterial blood pressure (ABP) was invasively monitored in order to obtain mean arterial pressure (MAP), and cerebral perfusion pressure (CPP) was calculated with the transducer placed at the tragus level.

ICPi was measured by means of either an intraparenchymal fiberoptic transducer (Codman, Johnson & Johnson Medical Ltd., Raynham, MA, USA, or Camino Laboratories, Integra LifeSciences, San Diego, CA, USA, RAUMEDIC) placed in the non-dominant frontal lobe, or a catheter inserted into the brain ventricles and connected to an external pressure transducer and drainage system (Codman, Johnson & Johnson Medical Ltd., Raynham, MA, USA).

### Study population

Sixteen centers in seven countries, chosen for their high volume of brain-injured patients and for their expertise in the field of TCD monitoring, enrolled a convenience sample of adult (≥ 18 years) critically ill neurological patients who required invasive ICP monitoring, either using intraventricular, intraparenchymal or post-craniotomy/postsurgical catheters, for any of the following diagnostic categories: traumatic brain injury (TBI), subarachnoid hemorrhage (SAH), intracerebral hemorrhage (ICH) and ischemic stroke (IS).

Patients were excluded if they had an inaccessible or poor acoustic ultrasound window, cardiac or vascular disease which had the potential of causing hemodynamic variations affecting the TCD measurement (i.e., severe arrhythmia, severe cardiac valvular stenosis, moderate or severe cerebral vasospasm), or a craniotomy or craniectomy performed prior to the first time frame readings. Patients were also excluded if they required treatment for suspected intracranial hypertension or manipulation of arterial blood pressure between the first ICP*tcd* estimation and immediately before or during the first ICP*i* measurements in TIME 1.

### Ethics approval

For the coordinating center of Brescia, Italy, the ethics committee approval was obtained on July 17, 2017 (NP-2762). The study approval was also obtained at each site from local regulatory boards before the study started. Written informed consent was obtained by family representatives. In the Italian centers, the requirement of informed consent was waived, because relatives are not regarded as legal representatives of the patient in the absence of a formal designation. Written informed consent was requested from all surviving patients if, and as soon as, they regained their mental competency.

### Outcome measures

Intracranial hypertension in TBI patients was defined as ICP*i* above 22 mmHg and lasting for at least 10 min. Since definition of intracranial hypertension is not well established in SAH, ICH and IS, we also assessed the accuracy of ICP*tcd* with ICP*i* above 20 mmHg and above 25 mmHg [17].

### Data collection

Given the focused nature of the study on diagnostic TCD accuracy, we collected a minimum number of parameters for parsimony, including demographic characteristics, cause of admission, neurological (Glasgow Coma Scale score) and neuroradiological severity (brain CT Marshall score), and type of device used to measure invasive ICP. De-identified data were extracted from electronic medical records and stored within the main database located at the coordinating center [18].

### Statistical analysis

Sample size was calculated using an exact method, assuming a true sensitivity of ICP*tcd* of 90% with lower acceptable confidence limit of 10%, resulting in a requirement of 147 cases. Assuming a prevalence of intracranial hypertension equal to 30%, the total sample size was 490 patients [19].

Continuous variables are presented as mean (standard deviation, SD) or median and interquartile range (IQR), and discrete variables as counts and percentages. Concordance correlation coefficient between ICP*tcd* and ICP*i* was calculated using variance components, estimated through linear mixed model to account for repeated measurements [19]. A Bland–Altman plot accounting for replicated measurements was fitted to estimate potential bias and limits of agreement. Since the main scope of the study was to assess the TCD as a real-practice screening method to exclude intracranial hypertension, we focused on negative and positive predictive values (NPV, PPV) and also calculated false omission rate (1 – NPV), representing the proportion of patients who have the condition among those with a negative test (i.e., intracranial hypertension ruled out). This is an important measure of a model’s misclassification of events [20]. Description of the TCD attributes (ICP*tcd*) relative to the reference standard (ICP*i*) was determined by calculating sensitivity, specificity, and the areas under a curve (AUC), for each ICP*i* threshold at each time frame (TIME 1, TIME 2 and TIME 3) and averaged over all three time points (*T*_1,2,3_). 95% confidence intervals (CI) were computed using bootstrapping (*B* = 2000). The best ICP thresholds were computed using the Youden criterion [21]. In case of missing or indeterminate index test or reference standard results, patients were excluded from the analysis. All tests were two-sided, and a *p* value of 0.05 was considered as the threshold for significance. The R software was used for statistical analysis (version 3.2.5, Free Software Foundation, Inc., Boston, MA, USA) [22]. Negative likelihood ratio (LR −) was used to evaluate the performance in ruling out the condition in the presence of a negative test (ICPtcd).

## Results

### Study population

**Fig. 2:**
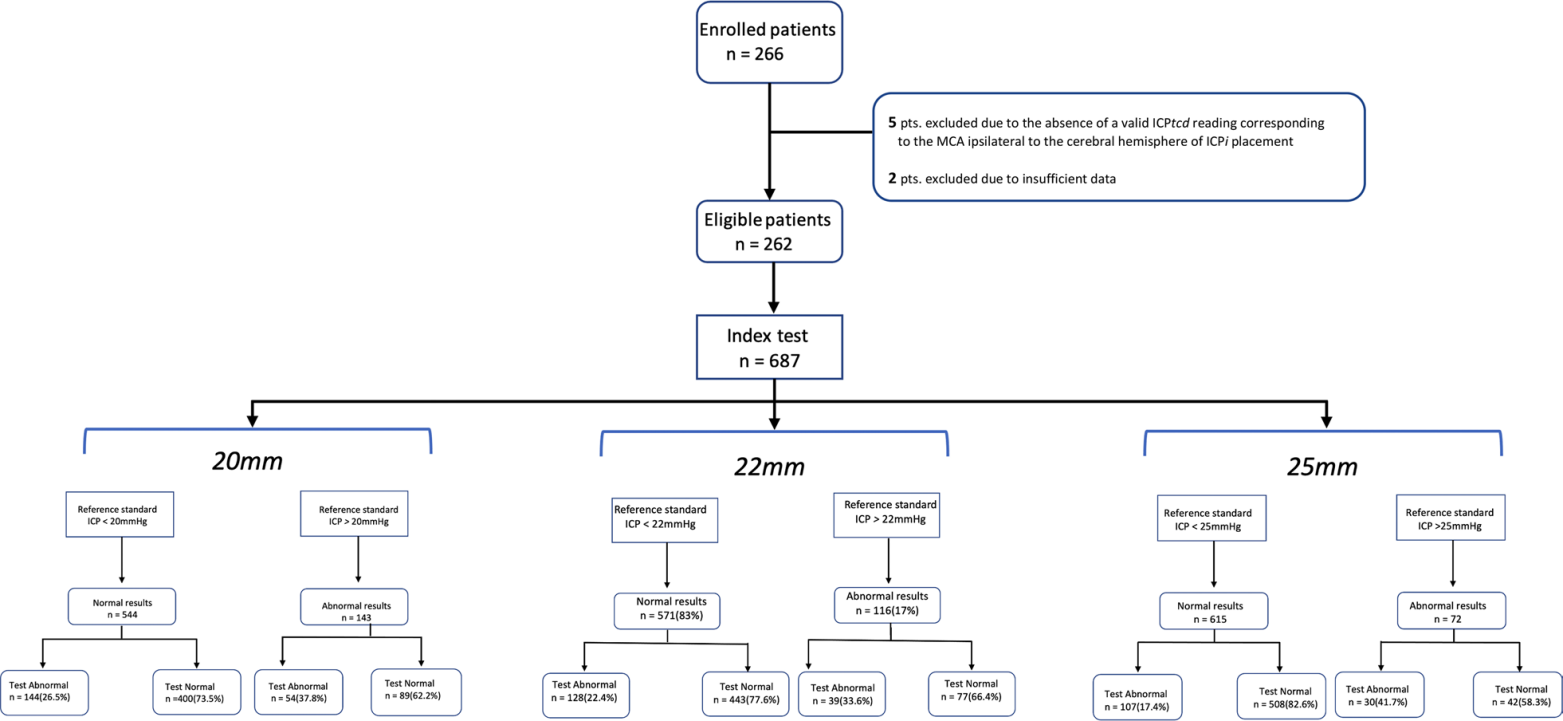
Patient recruitment plus the Standards for Reporting of Diagnostic Accuracy (STARD) flowchart

Recruitment started on July 2017 and was interrupted April 2020 after 266 patients were enrolled given the rapid reduction in the recruitment rate from late February 2020 onward when cases of COVID-19 started to rise quickly in western countries. The number of patients recruited per center is presented in Additional file 2: Figure S1. Of 266 patients recruited, 178 (70.4) were males and 75 (29.6) were females. The mean age was 65 years (SD 24.8). Four patients were excluded due to protocol violation (missing or indeterminate index test or reference standard results), leaving 262 patients and a total of 687 paired ICP*tcd* and ICP*i* measurements performed (Fig. 2).

The main characteristics of the study population are presented in Table 1.

**Table 1:** General characteristics of the study population

Type of brain injury	N° patients (%)	*Score*	*Median (IQR)*	Type of device for ICP measurement N° (%)	
TBI	135 (53.3)	GCS	6 (4–7)	**EVD**	*12 (8.9)*
		MARSHALL	3 (2–4)	**IP**	*113 (84)*
				**SD**	*10 (7.4)*
ICH	36 (14.2)	GCS	7 (4–8)	**EVD**	*26 (72)*
				**IP**	*10 (2.7)*
				**SD**	*0 (0)*
SAH	75 (29.6)	WFNS	4 (2–5)	**EVD**	*64 (85)*
		FISHER	4 (3–4)	**IP**	*11 (13)*
				**SD**	*0 (0)*
IS	7 (2.8)	NHSS	21 (20–23)	**EVD**	*3 (42.8)*
				**IP**	*1 (14)*
				**SD**	*3* (*42.8)*

Bold abbreviations in the first column indicate type of brain injury. TBI, traumatic brain injury; SAH, subarachnoid hemorrhage; ICH, intracerebral hemorrhage; IS, ischemic stroke; GCS, Glasgow Coma Scale; WFNS, World Federation of Neurosurgical Societies; NHSS, National Health Stroke Scale. Bold abbreviations in the last column indicate the type of invasive intracranial pressure monitoring device. EVD, external ventricular drain; IP, intraparenchymal; SD, subdural. Italic values indicate number, and percentage, of devices inserted

All 262 patients enrolled were investigated with both the index test (ICP*tcd*) and the reference standard (ICP*i*) with no protocol violation (Fig. 2, Additional file 3: Appendix 2).

ICP*tcd* versus ICP*i.*

The mean ICP*i* was 13.8 (SD 9.7) mmHg and intracranial hypertension (> 22 mmHg) occurred in 87 patients (33.2%). ICP*tcd* was higher than ICP*i* in 412 measurements (60%); details regarding intracranial hypertension according to different ICP thresholds (> 20 mmHg, > 22 mmHg and > 25 mmHg) are reported in Additional file 4: Table S1. NPV was high, 91.3%, 95.6% and 98.6% for ICP*i* threshold, respectively, of 20 mmHg, 22 mmHg and 25 mmHg, and 1-NPV was low, ranging from 9 to 2%, indicating high discriminant accuracy of ICP*tcd* in excluding intracranial hypertension with few model misclassifications of events. Data regarding the PPV, NPV, 1-NPV, sensitivity, specificity, accuracy, the positive and negative likelihood ratios and AUC for the three different ICP*i* thresholds during each separate and averaged time frame are reported in Table 2 and Additional file 5: Figure S2.

**Table 2:** Descriptors of diagnostic accuracy of intracranial pressure measured with transcranial Doppler (ICPtcd) compared to invasive ICP measurement (ICPi) at three different ICP thresholds and three time frames (T1, T2 and T3)

ICP thresholds	Time frames of ICP measurements			Averaged values of all three time frames
Above 20 mmHg	T1	T2	T3	
Optimal ICP threshold, mmHg	16.5 (15.5–24.5)	21.5 (16.5–27.0)	21.5 (13.5–25.5)	21.5 (12.5–24.5)
Sensitivity, %	75.8 (51.6–87.1)	62.5 (42.5–80.0)	65.9 (43.9–92.7)	65.1 (44.2–90.7)
Specificity, %	66.0 (54.7–86.8)	79.4 (65.8–90.5)	78.4 (42.7–88.6)	74.4 (40.2–88.1)
PPV, %	47.6 (40.0–63.0)	38.4 (28.0–53.7)	37.9 (25.4–51.6)	32.6 (22.7–46.3)
NPV, %	87.4 (81.2–92.9)	91.5 (88.1–95.0)	90.9 (87.1–96.4)	91.3 (88.2–95.7)
1-NPV, %	12.6 (7.1–18.8)	8.5 (5.0–11.9)	9.1 (3.6–12.9)	8.7 (4.3–11.8)
Accuracy, %	69.7 (61.5–79.2)	76.6 (66.5–84.5)	75.2 (50.9–82.7)	72.5 (48.5–82.1)
LR +	2.2 (1.1–6.6)	3.0 (1.2–8.4)	3.0 (0.8–8.2)	2.5 (0.7–7.6)
LR −	0.4 (0.1–0.9)	0.5 (0.2–0.9)	0.4 (0.1–1.3)	0.5 (0.1–1.4)
AUC	73.3 (65.7–80.8)	69.0 (58.4–79.7)	73.0 (64.6–81.4)	71.5 (63.1–80.0)

	T1	T2	T3	Average
*Above 22 mmHg*				
Optimal ICP threshold mmHg	16.5 (15.5–23.5)	20.5 (16.5–25.5)	21.5 (14.5–25.5)	20.5 (15.5–29.5)
Sensitivity, %	86.8 (62.3–96.2)	71.0 (48.4–87.1)	75.0 (53.1–93.8)	70.4 (40.7–92.6)
Specificity, %	66.1 (57.1–86.9)	77.9 (62.0–89.9)	75.8 (51.5–87.6)	71.9 (51.9–94.0)
PPV, %	45.0 (38.9–62.7)	32.7 (23.1–48.2)	32.6 (22.7–47.2)	23.0 (16.2–50.0)
NPV, %	93.6 (87.6–98.1)	94.6 (91.9–97.6)	94.8 (91.7–98.4)	95.6 (93.1–98.5)
1-NPV, %	6.4 (1.9–12.4)	5.4 (2.4–8.1)	5.2 (1.6–8.3)	4.4 (1.5–6.9)
Accuracy, %	71.5 (64.3–82.8)	77.0 (64.4–86.2)	75.2 (57.1–84.5)	71.8 (55.3–89.7)
LR +	2.6 (1.5–7.3)	3.2 (1.3–8.6)	3.1 (1.1–7.6)	2.5 (0.8–15.5)
LR −	0.4 (0.1–0.8)	0.4 (0.1–0.8)	0.3 (0.1–0.9)	0.4 (0.1–1.1)
AUC	80.2 (73.8–86.7)	74.1 (63.3–84.8)	77.0 (68.4–85.6)	75.6 (65.6–85.5)
*Above 25 mmHg*				
Optimal ICP threshold mmHg	22.5 (18.5–24.5)	19.5 (17.5–28.5)	17.5 (14.5–29.5)	25.5 (19.5–29.5)
Sensitivity, %	78.1 (59.4–90.6)	81.0 (52.4–95.2)	78.9 (47.4–100.0)	81.2 (56.2–100.0)
Specificity, %	76.7 (63.0–86.8)	72.5 (63.3–91.7)	69.6 (46.4–91.3)	84.1 (63.8–95.1)
PPV, %	35.5 (26.5–48.1)	22.2 (16.3–42.9)	18.6 (13.0–38.9)	23.1 (13.1–48.3)
NPV, %	95.2 (92.0–98.0)	97.3 (94.7–99.3)	97.2 (94.5–100.0)	98.6 (96.8–100.0)
1-NPV, %	4.8 (2.0–8.0)	2.7 (0.7–5.3)	2.8 (0.0–5.5)	1.4 (0.0–3.2)
Accuracy, %	76.5 (65.6–84.6)	73.2 (64.8–89.1)	70.4 (50.0–88.5)	83.6 (65.3–93.5)
LR + (sens/1 − spec)	3.4 (1.6–6.9)	2.9 (1.4–11.5)	2.6 (0.9–11.5)	5.1 (1.6–20.5)
LR − (1 − sens/spec)	0.3 (0.1–0.6)	0.3 (0.1–0.8)	0.3 (0.0–1.1)	0.2 (0.0–0.7)
AUC	78.4 (69.7–87.0)	76.2 (64.2–88.2)	75.1 (63.7–86.5)	83.2 (71.4–94.9)

PPV, positive predictive value; NPV, negative predictive value; LR, likelihood ratio; AUC, area under the curve. Numbers indicate the estimated values via bootstrap (95% confidence interval).

Regarding negative likelihood ratio (LR-), this has a constant value of 0.3 across time, corresponding to a moderate performance in ruling out the condition, with an approximate 25% reduction in condition probability after a negative test (ICP*tcd*).

The best measured ICP*tcd* threshold was 20.5 mmHg, corresponding to a sensitivity of 0.70 (0.41–0.93) and a specificity of 0.76 (0.70–0.81) with an AUC of 0.76 (95% CI 0.66–0.85) to predict intracranial hypertension.

**Fig. 3:**
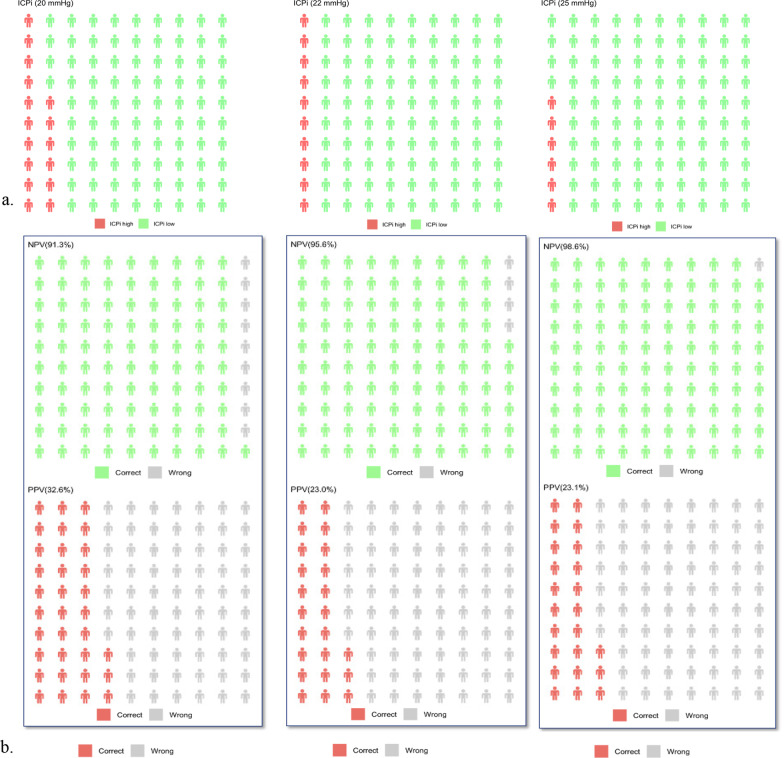
Percentages of patients with intracranial hypertension (IH) and negative (NPV) and positive (PPV) predictive values at the three intracranial pressure (ICP) thresholds. **a** Percentage of patients with ICP above (red silhouette indicating IH) and below (green silhouette indicating normal ICP) the given ICP thresholds of 20, 22 and 25 mmHg. **b** NPV and PPV (%) of the averaged time frames (*T*_1,2,3_) at the three ICP thresholds (20, 22 and 25 mmHg). NPV = green silhouette indicating true negatives and grey silhouette indicating false negatives. PPV = red silhouette indicating true positives and grey silhouette indicating false positives

The percentage of patients with ICP above and below the given thresholds of 20, 22 and 25 mmHg and NPVs and PPVs of the averaged time frames (*T*_1,2,3_) at the same thresholds are presented in Fig. 3.

Furthermore, we also evaluated if there were any discrepancies regarding the descriptors of diagnostic accuracy of ICP*tcd* compared to ICP*i* at three different ICP*i* thresholds (20, 22 and 25 mmHg) in TBI versus non-TBI non-TBI patients, and found no substantial differences (Additional file 6: Table S2).

**Fig. 4:**
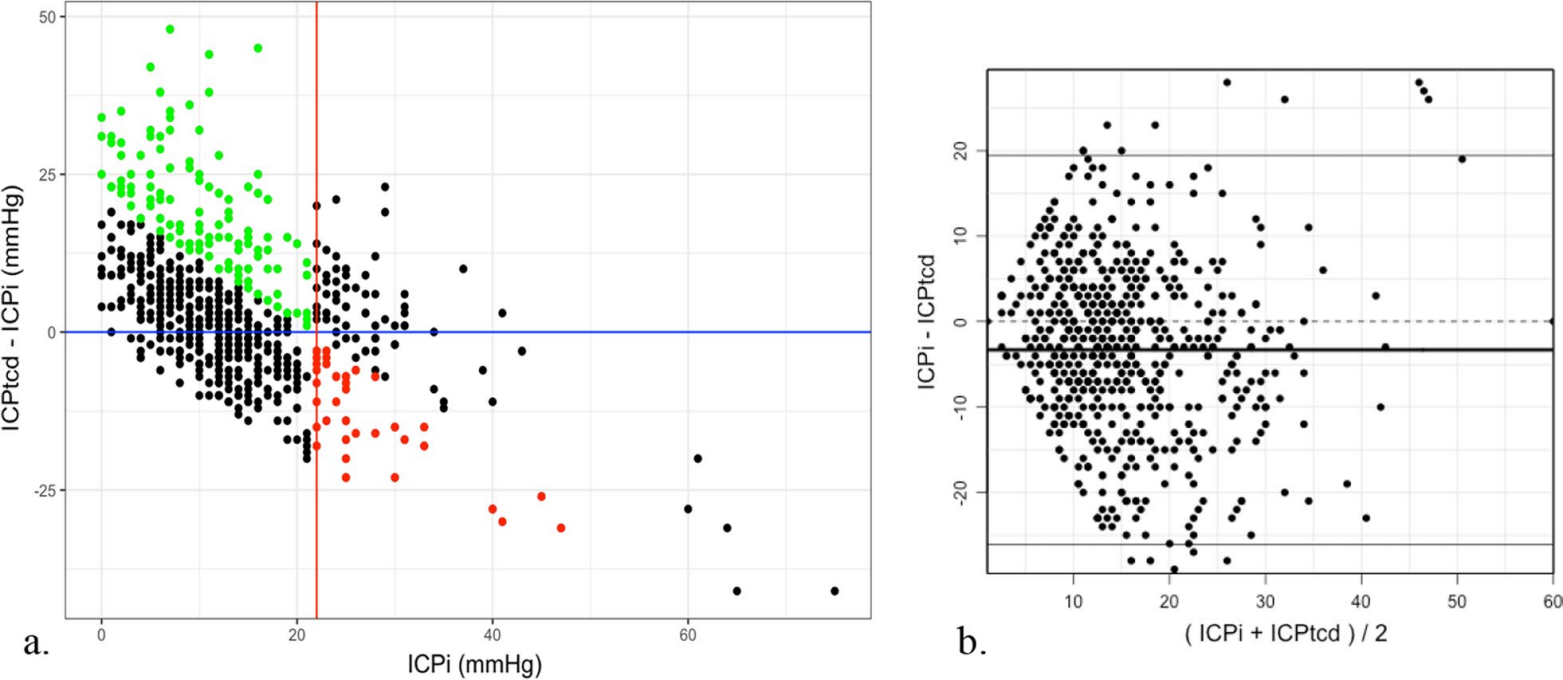
**a** Distribution of differences between paired ICPtcd and ICPi measurements as a function of ICPi. Black points represent concordant measurements (either true positive and true negative cases); green points indicate ICPi < 22 and ICPtcd ≥ 22 (false positive measurements); red points indicate ICPi ≥ 22 and ICPtcd < 22 (false negative cases). **b** Bland–Altman analysis yielded a mean bias (ICP*tcd*–ICPi) of − 3.3 mmHg with an agreement range comprised between − 26.1 and + 19.5

Agreement between ICP*tcd* and ICP*i*, estimated using concordance correlation coefficient, was 33.3% (25.6–40.5%) (Fig. 4a). Bland–Altman analysis yielded a mean bias (ICP*tcd*–ICP*i*) of -3.3 mmHg with limits of agreement comprised between −26.1 mmHg and + 19.5 mmHg (Fig. 4b, Additional file 7: Figure S3).

When comparing *relevant clinical variables* (CPP, TBI vs non-TBI and GCS) in patients with concordant or discordant ICP*i* and ICP*tcd* readings, we found that CPP showed a significant trend, with lower values in false negative and higher values in false positives, compared to concordant readings. All measurements were considering the average value over the three timings (Additional file 8: Table S3).

## Discussion

In this prospective, international, multicenter study, we found that TCD reliably excluded intracranial hypertension in a variety of acute neurological injuries. Since TCD application is noninvasive, our results may have an impact on daily practice in situations where invasive ICP measurement is impractical or is not feasible. Of those cases identified as having normal ICPtcd, 91% had normal ICPi using a threshold of > 20 mmHg, 96% using a threshold > 22 mmHg and 99% using a threshold of > 25 mmHg. ICP*tcd* was estimated at the three separate time points without significant changes of the test performance over time, indicating that ICP*tcd* estimation of ICP*i* was not time dependent. At TIME1, ICP*tcd* was measured immediately before skull drilling for intracranial insertion of invasive monitoring, thus reproducing the real situation of TCD use in clinical practice in a patient with an intact skull for whom only clinical information is available.

To date, this study is the first to demonstrate the value of ICP*tcd* as a screening test to exclude intracranial hypertension in patients with acute brain injury. We found four single-center observational studies [23–26], of which three were prospective [24–26] and only one was multicenter study [15]. A recent meta-analysis [17] found a sixth retrospective, single-center study [27]. Accuracy of TCD was reported in terms of the screening test’s attributes relative to the reference standard, mainly as ROC analysis and AUC, and hence the usefulness of the test in clinical practice was not specifically addressed. Only one study in 21 patients with acute liver failure reported that an ICP*tcd* > 18.55 mmHg had a NPV of 100% (95% CI 74%-100%) and a PPV of 56% (95% CI 34%-75%) for the detection of concurrent ICP*i* > 20 mmHg. [28] The authors concluded that ICP*tcd* may in fact be most useful as a screening tool to exclude intracranial hypertension. Two recent prospective studies found contrasting results in terms of diagnostic accuracy of TCD, with one study reporting good diagnostic test accuracy with 81% sensitivity and 70% specificity and the other reporting 0% sensitivity and 74.4% specificity for ICP*tcd* to detect ICP*i* > 20 mm Hg mmHg [28, 29]. None of these two studies assessed the predictive ability of TCD in excluding intracranial hypertension.

**Fig. 5:**
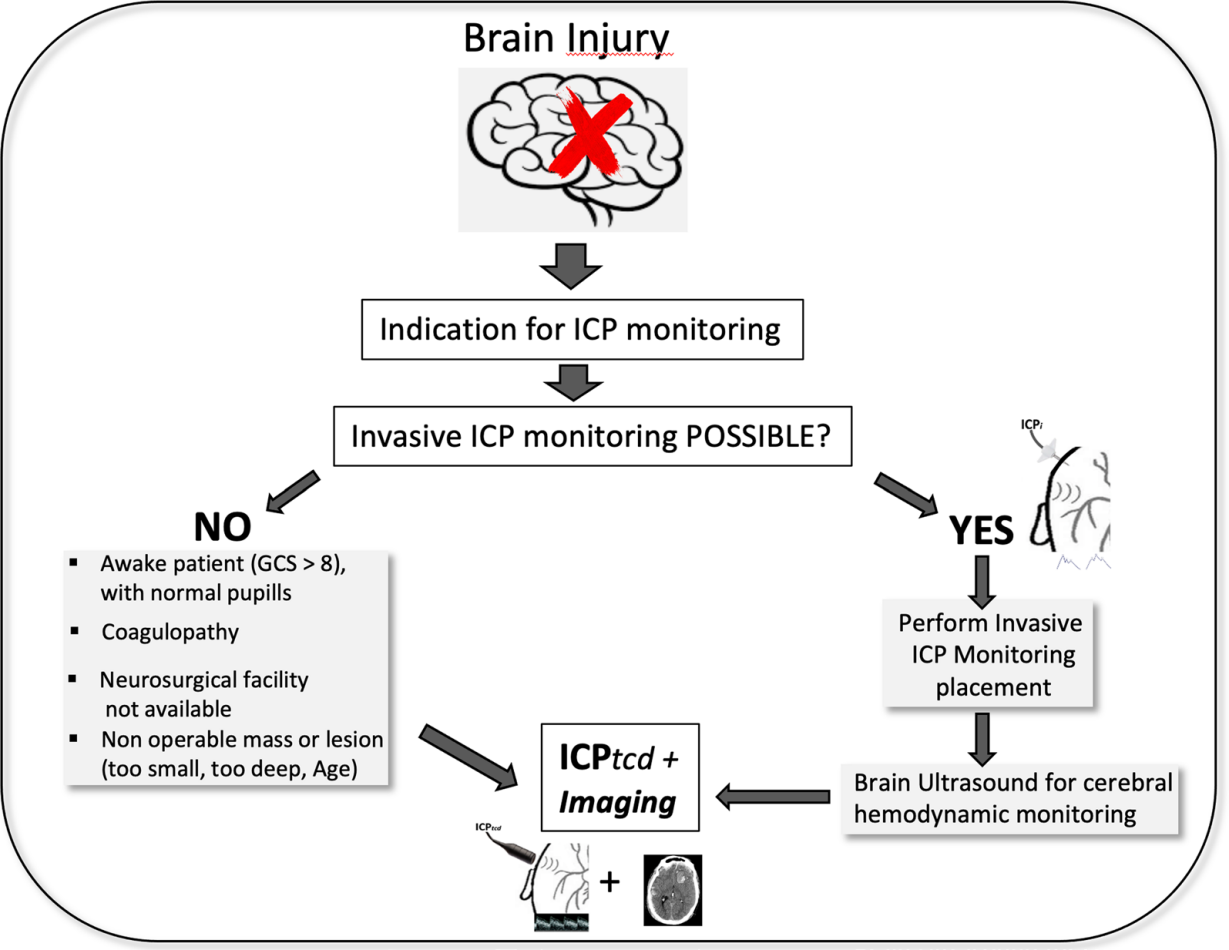
Algorithm for noninvasive intracranial pressure monitoring through the use of transcranial Doppler (ICP*tcd*). Once indication for ICP monitoring is decided, application of the invasive method (gold standard) is evaluated: If **NO** (invasive ICP monitoring not possible) for the presence of one or more of the reasons provided, ICP*tcd* associated with brain imaging should be performed. *The result should not act as a deterrent for transferal towards a hospital with a Neurosurgical facility or to perform further brain imaging studies, instead ICPtcd should be used as an adjunct capable of providing valuable information for the Clinician; if **YES** (invasive ICP monitoring possible), then burr hole and insertion of a catheter within the brain parenchyma should be performed. *However, brain ultrasound with transcranial Doppler may be useful in order to obtain surrogate information regarding brain hemodynamics through the evaluation of cerebral blood flow velocity

There is general agreement that sensitivity and specificity, which are important descriptors of the accuracy of a diagnostic test, should be applied only in the context of describing the screening test’s attributes relative to a reference standard [30]. With sensitivity and specificity, the main question addressed is: “Is the screening test adequate?”. The problem addressed is the fundamental “credentials” of the screening test [13]. However, if the main question is: “Do the results on the screening test correspond to the results on the reference standard?”, as should be in the context of real-practice screening, it is the screening test that is being assessed, and positive and negative predictive values are more appropriate and informative. In fact, diagnostic testing is used because clinicians want to know the probability of the condition existing or not existing. Since clinicians make decisions based on diagnostic test results and not necessarily on results of reference standards, positive and negative predictive values can be more important than sensitivity and specificity for clinical practice. If the test has a high NPV, there is a high probability that a patient does not have the pathological condition being investigated when the patient has a negative test. This is particularly relevant if the condition to be excluded is a life-threatening, time-dependent condition, such as acute intracranial hypertension, and invasive ICP monitoring is not available or is impractical. A recent study found that automated pupillometry performed in 23 patients with spontaneous intracerebral hemorrhage had high NPV in excluding intracranial hypertension [31]. In patients with acute liver failure, elevated ICP is an important cause of death and disability; ICP*tcd* may be helpful in these cases, because coagulopathy, which is always present, increases the risk of intracranial hemorrhage following monitor insertion [27]. During triage of polytrauma patients, ICP*tcd* may be helpful in prioritizing treatment when extracerebral lesions are also involved. In the general ICU population, especially in cardiac arrest patients or in metabolic coma and liver failure, ICP*tcd* can be an important tool for the early diagnosis and treatment of cerebral complications [32]. Another field where a noninvasive ICP monitoring technique would be most useful would be developing countries where access to hospitals with resources for invasive ICP monitoring is scarce [33, 34]. Finally, in patients at risk for intracranial hypertension, repeated TCD assessment is easily implemented and may represent the primary monitoring method if ICP*tcd* remains low. If ICP*tcd* indicates intracranial hypertension, further diagnostic modalities, including a prompt decision for invasive ICP monitoring, should be considered. A simple algorithm for application of ICP*tcd* is provided in Fig. 5.

Of those measurements identified as having intracranial hypertension at ICPtcd, 67.4% (53.7–77.3) had intracranial hypertension at ICPi using a threshold of > 20 mmHg, 77.0% (50.0–83.8) using a threshold > 22 and 76.9% (51.7–86.9) using a threshold of > 25 mmHg. Reasons for this duality of ICP*tcd,* an excellent screening test to exclude intracranial hypertension and a poor screening test to confirm it, remain speculative. Unlike a pressure transducer, TCD is a measurement of blood flow velocity and not a direct measurement of pressure and is greatly influenced by the ratio between FVd and FVm: the lower the ratio, the higher the ICP*tcd* value. A low ratio may occur in the presence of true intracranial hypertension due to external vessel compression and increased resistance, but also in the case of normal ICP*i* with decreased MAP and altered cerebrovascular autoregulation. In both cases, the transmural pressure (i.e., the “extra-mural” ICP minus the intramural arterial pressure) increases with a disproportionate reduction in FVd compared to FVm and, hence, a reduction in their ratio.

### Limitations

Some study limitations are worth considering: First, as mentioned previously, recruitment stopped after 266 patients were enrolled; therefore, we did not reach the target sample size of 490 patients. Nevertheless, we believe that the number of patients recruited was sufficient enough to perform an adequate statistical analysis of our study aim; second, we do not have an exact number of patients screened among the 16 centers who took part in this study; third, we enrolled patients with different types of brain injury, including IS, SAH and ICH, for which ICP thresholds are not as well defined as for TBI; fourth, the study was unblinded and therefore correct reporting of paired ICPi and ICPtcd may have been imprecise; fifth, the incidence of intracranial hypertension was lower than in other series, which may limit the generalizability of results to other populations with more severe disease; sixth, hemodynamic instability during critical illness due to extracranial causes (sepsis, hemorrhage, etc.) may influence the correlation between true ICP*i* and ICP*tcd* leading to a probable disassociation between the two; and finally, as per study design, the patients’ outcome was not assessed, and hence, the impact of using TCD in reducing morbidity and mortality remains unanswered.

Despite the study limitations, the results in this study confirm the results obtained from our previous multicenter pilot IMPRESSIT-1 study, which showed a NPV of 100% in excluding intracranial hypertension at ICP thresholds of > 20 and > 22 mmHg, and at the best threshold value of 24.8 mmHg. [19] TCD screening test attributes relative to the reference standard were better than in the current study, with 100% sensitivity and 70% (95% CI 54.5–84.8%) specificity at ICP thresholds of > 20 and > 22 mmHg, and at the best threshold value of 24.8 mmHg. The technique remains operator-dependent, and among the 16 centers involved there may have been heterogeneity regarding the skills of the various sonographers. It must be stressed that as for any monitoring tool, correct training should be performed before implementation of the technique.

Nevertheless, TCD retained excellent screening performances, suggesting that its use to exclude intracranial hypertension in everyday clinical practice, where the presence of multiple sonographers represents a common situation, is feasible.

## Conclusion

This study demonstrates that ICP*tcd* has a high NPV compared to ICP*i* in a variety of acute neurological conditions. As such, TCD may prove useful to exclude intracranial hypertension in situations where invasive methods cannot be used or are not available, and to screen at-risk patients for potential invasive ICP monitoring.

## Supplementary Information

**Additional file 1.** STARD checklist.

**Additional file 2.** **Figure S1.** Total number of patients recruited per center.

**Additional file 3.** Study Protocol.

**Additional file 4.** **Table S1.** Number of measurements of invasive intracranial pressure (ICP*i*) and trans-cranial Doppler (TCD)-estimated ICP (ICPtcd) at the three time-frames (T_1_, T_2_ and T_3_) in relation to the three ICP different thresholds (> 20 mmHg, > 22 mmHg and > 25 mmHg).

**Additional file 5.** **Figure S2.** Forest plot indicating the areas under a curve (AUC), sensitivity and specificity for each time frame (T_1_, T_2_, T_3_).

**Additional file 6.** **Table S2.** Descriptors of diagnostic accuracy of intracranial pressure measured with transcranial doppler (ICP*tcd*) compared to invasive ICP measurement (ICP*i*) at three different ICP*i* thresholds (20, 22 and 25 mmHg) in Traumatic (TBI) vs non-TBI (non-TBI) brain injury. PPV = positive predictive value. NPV = negative predictive value. LR = Likelihood Ratio. AUC = area under the curve.

**Additional file 7.** **Figure S3.** Bland-Altman analysis for each time frame.

**Additional file 8.** **Table S3.** Comparison of relevant clinical variables (CPP, TBI vs non-TBI and GCS) in patients with concordant or discordant ICP*i* and ICP*tcd* readings at the three ICP*i* thresholds, (20, 22 and 25 mmHg).CPP showed lower values in the False Negatives and higher values in False Positives compared to Concordant readings. All measurements considered the average value over the the three time-frames (T_1–3_).

## Data Availability

All data are available to the study participants and to the reviewers when and if requested.

## Abbreviations

ICU: Intensive care unit
AUC: Area under the receiver operating characteristic curve
ICP: Intracranial pressure
ICP*i*: Invasive intracranial pressure
ICP*tcd*: Transcranial Doppler measurement of intracranial pressure
TCD: Transcranial Doppler
CPP: Cerebral perfusion pressure
ABP: Arterial blood pressure
MABP: Mean arterial blood pressure
SAH: Subarachnoid hemorrhage
ICH: Intracerebral hemorrhage
TBI: Traumatic brain injury
IS: Ischemic stroke
ROC: Receiver operating characteristic
NPV: Negative predictive value
PPV: Positive predictive value
CI: Confidence interval
