# Supplementary figures and images for: Transcranial Doppler as a screening test to exclude intracranial hypertension in brain-injured patients: the IMPRESSIT-2 prospective multicenter international study
Source: Crit Care. 2022 Apr 15;26:110. doi: 10.1186/s13054-022-03978-2 (PMC9012252; doi:10.1186/s13054-022-03978-2)

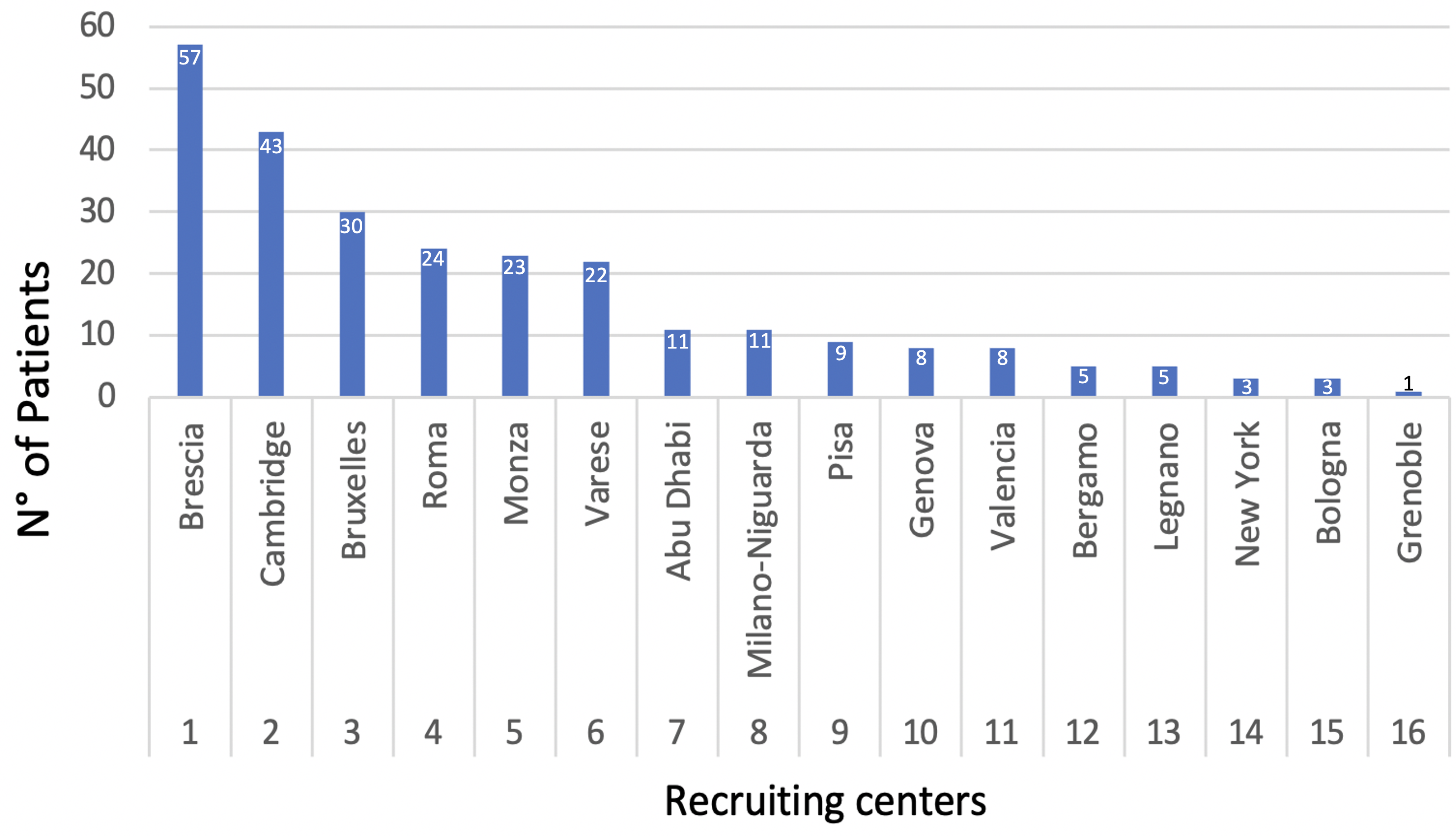


**Figure S1.** Total number of patients recruited per center.

Supplement: Supplementary file 2 — Additional file 2. Figure S1. Total number of patients recruited per center. [file 13054_2022_3978_MOESM2_ESM.docx]
