# Supplementary material for: Transcranial Doppler as a screening test to exclude intracranial hypertension in brain-injured patients: the IMPRESSIT-2 prospective multicenter international study
Source: Crit Care. 2022 Apr 15;26:110. doi: 10.1186/s13054-022-03978-2 (PMC9012252; doi:10.1186/s13054-022-03978-2)
Supplement: Supplementary file 3 — Additional file 3. Study Protocol. [file 13054_2022_3978_MOESM3_ESM.docx]

Trans-Cranial Doppler as a screening test to exclude intracranial hypertension in brain injured patients: the IMPRESSIT-2 prospective multicenter international study

**Study Protocol**


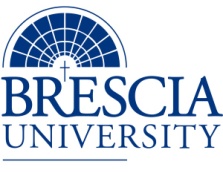
 [
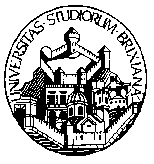
](http://www.unibs.it/)

**I**NVASIVE vs NON INVASIVE **M**EASUREMENT OF INTRACRANIAL **PRESS**URE

**I**N BRAIN INJURY **T**RIAL

**IMPRESSIT *2***

Table of contents

1 General information ……………………………………………………… page 2

1.1 Organization ……………………………………………………… page 3

1.2 Protocol summary …..……………………………………………. page 4

## 2 Rationale and aim of the study …………………………………………. page 5

## Study endpoints …………………………………………………… page 7

3.1Primary outcome …………………………………………………..

## 4 Study description ………………………………………………………… page 7

4.1 Study design ………………………………………………………

4.2 Sample size ………………………………………………………..

4.3 Expected study duration …………………………………………..

# 5 Study population ………………………………………………………… page 9

5.1 Inclusion criteria ……………………………………………………

5.2Exclusion criteria …………………………………………………..

# 6 Detailed study course …………………………………………………….. page 10

- 1. Flow diagram ………………………………………………………..

## 7 Organization ……………………………………………………………… page11

- 1. Collecting data …………………………………………………………
  2. Data management and archiving ………………………………………
     1. Data property ………………………………………………..
     2. Data control …………………………………………………
     3. Subsequent use of data ………………………………………
     4. Archiving ……………………………………………………

- 1. Sponsorship …………………………………………………………….

## 8 Ethics ……………………..…………………………………………………… page12

8.1 Approval by ethics committee …………………………………

8.2 Informed consent ………………………………………………

**9 Statistical analysis …………………………………………………………….** page12

**10 References ……………………………………………………………………..** pages13,14

**11 Appendix** page 14

11.1 Formula’s

**1 General information**

- 1. **Organisation**

| Principle Investigator | - Dr. Frank Rasulo: Neuro Critical Care, Azienda Ospedaliera Spedali Civili di Brescia, University of Brescia, (Italy) |
| --- | --- |
| Steering committee | - Soojin Park: Neuro Critical Care, New York Presbyterian Hospital, Columbia University, New York (USA) - Chiara Robba: Neuro Critical Care, Addenbrooke’s NHS trust, Cambridge University, (UK) - Fabio Silvio Taccone: Université Libre de Bruxelles , Hôpital Erasme, Brussels (Belgium) - Pierre Bouzat: Centre Hospitalier Universitaire Grenoble Alpes, Pôle Anesthésie Réanimation - Giuseppe Citerio: Neuro Critical Care, Azienda Ospedaliera San Gerardo di Monza, Università degli studi Milano-Bicocca, (Italy) |
| **Coordinating center** | Neuro Critical Care Unit, Department of Anesthesiology, Intensive care, and emergency Medicine, Spedali Civili Hospital of Brescia, University of Brescia, Italy |
| Study Statistician | Stefano Calza, Statistica medica e biometria, Statistician at the University of Brescia, Italy |
| **Data Management for IMPRESSIT-2 study purposes** | Study coordinators |

- 1. **Protocol summary**

| Title of the study | **Discriminant accuracy of Trans-Cranial Doppler to Exclude Intracranial Hypertension in Brain Injured Patients: The IMPRESSIT-2 Prospective Multicenter International Study** |
| --- | --- |
| **Primary outcome** | The NPV, PPV, sensitivity and specificity of ICPtcd estimation in excluding true intracranial hypertension, as measured by the gold standard invasive monitoring technique (ICPi). |
| **Design** | Prospective, observational, non-interventional, non-randomized, non-profit, international, multicenter, clinical study |
| Target population | **Inclusion criteria**   - Brain injury (Traumatic Brain Injury, Subarachnoid Hemorrhage, Intracerebral Hemorrhage, Ischemic stroke); - Patients requiring invasive ICP monitoring (Intraparenchymal, post-craniotomy or post-surgical, Intra-ventricular); - Age > 18 yrs.   **Exclusion criteria**   - inaccessible or poor acoustic ultrasound window, - a cardiovascular disease causing hemodynamic variations affecting the TCD reading (severe arrhythmia, severe cardiac valvular stenosis, moderate or severe vasospasm), - Patient already with craniotomy or craniectomy before first time frame 1°, - any treatment for suspected intracranial hypertension or manipulation of arterial blood pressure intervening between the non-invasive ICPtcd measurements and the insertion of invasive ICP (ICPi) measurement. |
| **Sample size** | 490 patients; (described in section 4.2) |
| **Interventions** | All patients included will have evaluation of non-invasive ICP monitoring through use of TCD (ICPtcd). ICPtcd will be calculated with the standard formula explained previously for non-invasive estimation of cerebral perfusion pressure (CPP)[Appendix11.1]*. (described in section 4.1). |
| **Study duration** | 24 months |

**2 Rationale and aim of the study**

As part of intensive treatment of brain injury, intracranial pressure (ICP) should be controlled when the cerebral perfusion pressure (CPP) falls below 70mmHg (depending on the cause of injury) and/or the ICP is greater than 22mmHg for > 10 min. Intracranial hypertension (ICHP) occurs in approximately 40% of all patients with severe traumatic brain injury and is not infrequent in patients with non traumatic brain injury, such as subarachnoid hemorrhage (SAH), spontaneous intracerebral hemorrhage (ICH), and ischemic stroke (IS), and the presence of ICHP and inadequate CPP has been correlated to bad outcome. Therefore, measures to monitor ICP and CPP should be instituted as soon as possible in patients with severe brain injury.

*Monitoring.* Monitoring of patients with brain injury (BI) is essential for the guidance and optimization of therapy. The rationale of monitoring is early detection and diagnosis of secondary brain insults, both systemic and intracranial and must comprise both general and specific neurologic monitoring. The Brain Trauma Foundation (BTF) guidelines for TBI management recommends that ICP should be monitored in all salvageable patients with severe TBI and an abnormal computed tomography (CT) scan. Based on physiological principles, potential benefits of ICP monitoring include earlier detection of intracranial mass lesion, guidance of therapy and avoidance of indiscriminate use of therapies to control ICP, drainage of cerebrospinal fluid (CSF) with reduction of ICP and improvement of CPP, and determination of prognosis. Currently, available methods for ICP monitoring include epidural, subdural, subarachnoid, parenchymal, and ventricular locations. Historically, ventricular ICP catheter has been used as the reference standard and the preferred technique when possible. It is the most accurate, and reliable method of monitoring ICP [1]. Subarachnoid, subdural, and epidural monitors are less accurate. Unfortunately, all of the described methods are invasive, associated with a complication rate and are not inexpensive.

*Transcranial Doppler*. New methods have been developed in order to measure ICP non-invasively. Transcranial Doppler (TCD) for example, is a technique for monitoring cerebral hemodynamics, introduced by Aaslid et al in 1982. [4]. By using a low-frequency–pulsed Doppler of 2MHz over the acoustic window regions—where the skull bone is thin—or at the foramen magnum, it is possible to measure flow velocities in the basal cerebral arteries. A broad spectrum of usage scenarios for TCD have been proposed, including detection of vasospasm in SAH, detection of cerebral embolization, and arterial steno-occlusive disease, evaluation of collateral circulation, evaluation of recanalization, cerebrovascular autoregulation, detection of cerebral circulatory arrest, and for measuring ICP. [5,6]. The American Society of Neuroimaging’s Practice Guidelines Committee has developed standardized guidelines for TCD performance.[7,8]. TCD Technique and ICP In 1987, Klingelhofer and colleagues first described a relationship between increasing ICP and decreasing TCD-derived flow velocities and an increase in the Pourcelot index or resistance index (RI), defined as RI= (FVpeak sys_FVend dia)/(FVpeak sys). [9,10,11].

Aaslid *et al.* suggested that an index of CPP could be derived from the ratio of the amplitudes of the first harmonics of the ABP and the MCA velocity (detected by transcranial Doppler sonography) multiplied by mean flow velocity. Recently, a method for the non-invasive assessment of CPP has been reported, derived from mean arterial pressure multiplied by the ratio of diastolic to mean flow velocity. This estimator can predict real CPP with an error of less than 10 mm Hg for more than 80% of measurements. This is of potential benefit for the continuous monitoring of changes in real CPP over time in situations where the direct measurement of CPP is not readily available. [12].

# *Non-invasive CPP measurement.* A more complex method aimed at the non-invasive assessment of ICP has been introduced and tested by Schmidt *et al.* The method is based on the presumed linear transformation between arterial pressure and ICP waveforms. Coefficients of this transformation are derived from the database of real ABP and ICP recordings. Similar linear transformation is built, using the same database between flow velocity and arterial pressure. Then the model assumes linear relationship between arterial pressure and flow velocity and arterial pressure to ICP transformations. Multiple regression coefficients are calculated. [13-15].

#

# estimated-CPP: MAP x diastolicFV-mca / meanFVmca + 14

*Non-invasive ICP measurement.*

# Finally, ICP is calculated:

# non-invasive ICP = MAP - non-invasive CPP [Appendix **]

# 3 Study endpoints

##

3.1 Primary outcome: the negative predictive value, positive predictive value, sensitivity and specificity of ICPtcd estimation in excluding true intracranial hypertension, as measured by the gold standard invasive monitoring technique (ICPi) [with catheters inserted either into the brain parenchyma or ventricles].

**4 Study description**

##

4.1a Study design

*Non-invasive vs Invasive measurements.*

For each patient enrolled into the study, a total of three ICPtcd measurements will be performed in three different time frames, each of which will be compared to the corresponding ICPi for concordance.

- The first ICPtcd measurement (TIME 1) will be performed before ICPi placement (< 30 min.) and will be compared with the first ICPi reading once the probe is positioned. The need to reduce the time gap as much as possible between the two readings is motivated by the fact that ICP may be subjected to variations caused by ABP manipulation, cerebrospinal fluid (CSF) leakage during catheter placement and pharmacological treatment or fluctuations due to the evolving underlying brain injury.

-The second ICPtcd measurement (TIME 2) will be performed immediately after insertion of the ICPi probe and compared with the post-insertion ICPi reading. The ICPi measurement to be confronted with the ICPtcd estimated measurement for concordance must be read simultaneously.

- The third ICPtcd measurement (TIME 3) is performed between 2 and 3 hours following the second reading. The reason for this is to avoid any possible variations in systemic and cerebral hemodynamics caused by the ICPi device insertion itself, despite sedation. Therefore, performing the examination more than 2 hours post insertion should reduce the influence of the positioning maneuver on the readings. The ICPi measurement to be confronted with the ICPtcd estimated measurement for concordance must be read simultaneously.

In accordance with the present guidelines, intracranial hypertension is defined as an ICP above 22 mmHg, which remains so for at least 10 minutes and is not related to procedural pain [16]. Therefore, intracranial hypertension is considered such only when the ICPi reading meets this criteria.

However, when analysis will be performed the ICPtcd may be confronted to three different ICP values for concordance correlation (ICPi=20mmHg, ICPi=22mmHg, ICPi=25mmHg,).

*Patient sedation.*

Patient sedation for ICP bolt placement may consist of either bolus doses of sedatives and or analgesics and when necessary, neuromuscular blockade of choice, as long as these have been administered either before or after, and not between, the first concordance correlation. As mentioned previously this restriction is not necessary for the following two Time frames.

Mechanical ventilation should be targeted to maintain adequate oxygenation (SaO2 > 90%) and normocapnia (PaCO2 36–40 mmHg). Intravenous fluids and inotropic support (norepinephrine and/or epinephrine) are appropriate in order to achieve and maintain a sufficient cerebral perfusion pressure (CPP >60 mmHg). General management of the various types of brain injury (traumatic, hemorrhagic, or ischemic), as well as the definition of intracranial hypertension, should be in accordance to international guidelines [16]. Treatment of intracranial hypertension was based on a protocol-driven strategy which included optimization of arterial blood pressure and volemia, sedation, mild hyperventilation, and infusion of hyperosmolar fluids [17].

*Patient monitoring.*

Systemic hemodynamic monitoring will consists of invasive arterial blood pressure (ABP) from the radial artery, continuous electrocardiography and pulse oximetry. ICPi may be performed either by means of an intraparenchymal fiberoptic transducer or a catheter inserted into the brain ventricles and connected to an external pressure transducer and drainage systems (external ventricular drain).

For CPP calculation. the arterial transducer should be leveled at the height of the external auditory meatus of the ear, or tragus.

Cerebral blood flow velocity will be assessed using TCD sonography and should be performed by a selected group of experienced operators in order to reduce inter-operator variability.

The insonation technique is standard: a low frequency pulsed 2 MHz ultrasound probe to be placed over the acoustic temporal window for insonation of the M1/M2 section of the middle cerebral artery (MCA) at a depth ranging from 45 to 55 mm. The MCAs are to be insonated bilaterally; however, for ICPtcd measurements, the acoustic window ipsilateral to the side of ICP bolt placement is preferred. The Doppler parameters necessary for calculation on ICPtcd are:

# - Middle Cerebral Artery Diastolic Flow Velocity (FVd-mca)

# - Middle Cerebral Artery Mean Flow Velocity (FVm-mca)

During the three time frames measurement of CO_2_  crucial. At time frame 1° both ET CO_2_ and PaCO_2_ can be measured for calibration. However it is suggested to use the same measurement parameter for all three time frames, whether it be ET CO_2_ or PaCO_2_.

# *Throughout the trial all patients will receive standard clinical treatment according to the international guidelines for the treatment of various types of brain injury.

4.2 Sample size

Sample size was estimated using the approach suggested by Flahault et al. (2005) and H. Chu and S.R. Cole (2007), assuming a *true* sensitivity of the test (ICPtcd) of 90%, a prevalence of the disease (intracranial hypertension) equal to 30%, a power of 95% and a lower acceptable confidence limit of 10%.

We calculated an overall sample size of **490** patients

4.3 Expected study duration: at completion of the inclusion of 490 patients, roughly two years are expected to be recruited.

# 5 Study population

# 5.1 Inclusion criteria

# brain injury (Traumatic Brain Injury, Subarachnoid Hemorrhage, Intracerebral hemorrhage, Ischemic stroke);

# patients requiring invasive ICP monitoring (intraparenchymal, post-craniotomy or post-surgical, Intra-ventricular);

# age > 18 yrs.

# 5.2 Exclusion criteria

# - inaccessible or poor acoustic ultrasound window,

# - a cardiovascular disease causing hemodynamic variations affecting the TCD reading (severe arrhythmia, severe cardiac valvular stenosis, moderate or severe vasospasm),

# - Patient already with craniotomy or craniectomy before first time frame 1°,

# - any treatment for intracranial hypertension or manipulation of arterial blood pressure intervening between the non-invasive ICPtcd measurements and the invasive ICP (ICPi) measurement. (This is most likely to happen during time frame 1°). For this reason it is stressed that if the patient requires invasive ICPi monitoring to be performed in the operating room, than the non-invasive ICPtcd measurement should be performed just before the patient leaves the ICU (or if possible the ER) towards the OR for insertion. This would reduce the time during which the patient may be prone to ABP and ICP manipulation.

# 6 Detailed study course

#

Flow diagram


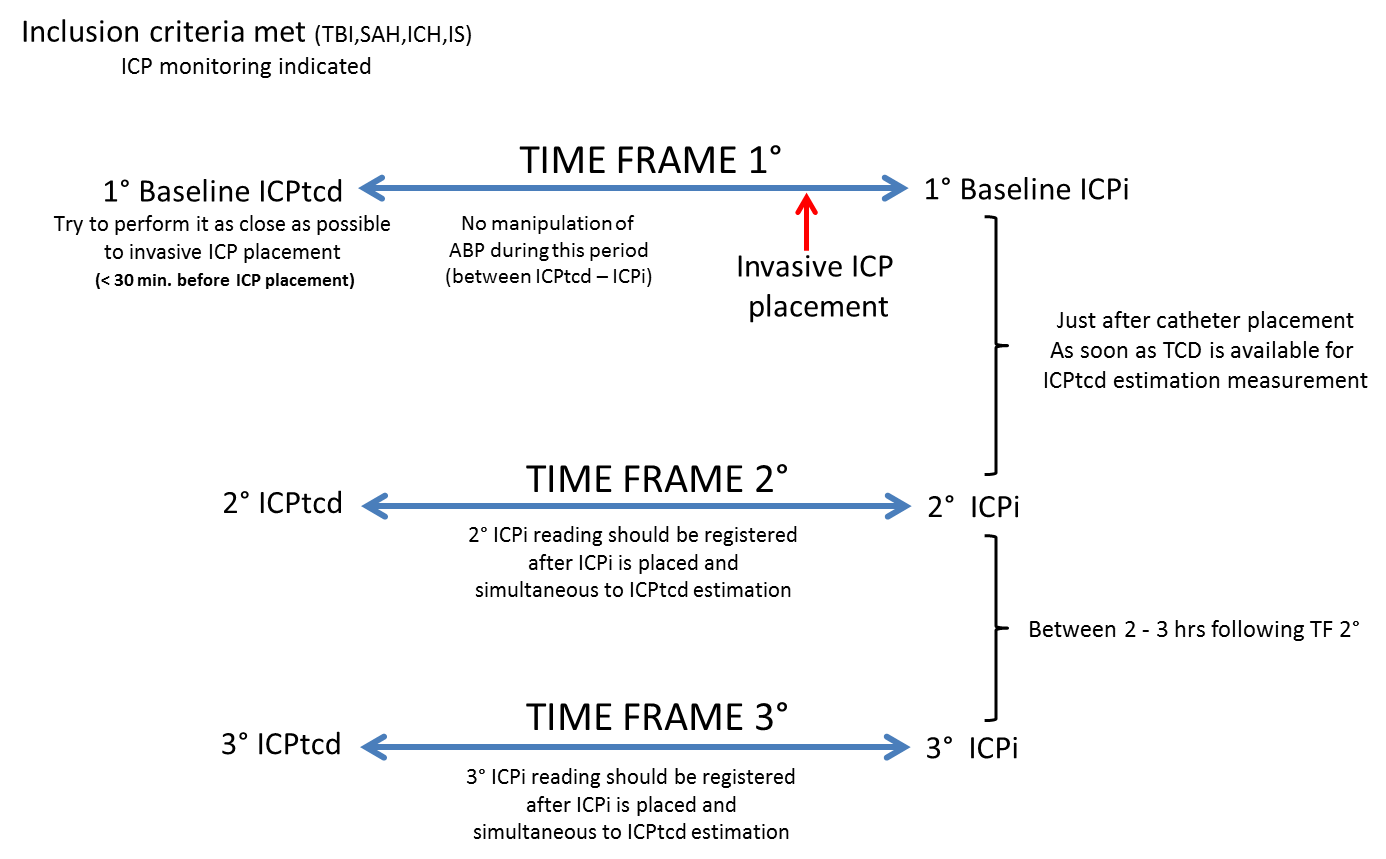


## 7 Organization

7.1 Collecting data

Data collection will be performed by the personal pertaining to each single center and entered into an on-line data collection program (RedCap), which will automatically be downloaded by the coordinating center for analysis.

7.2 Data management and archiving

7.2.1 Data property

Property of the data belongs to the coordinating center (Brescia).

7.2.2 Data control

Control of the data will be performed by the coordinating center.

7.2.3 Subsequent use of data

Initial use of the data belongs to the centers pertaining to the coordinating center until the data has been processed and analyzed for the first publication. Further use of the data for subsequent publications may be possible by part of the steering committee.

7.2.4 Archiving

Data will be archived by the coordinating center who will assume responsibility.

7.3 Sponsorship

No sponsorship nor funding.

## 8 Ethics and Consent

## Ethics approval for all participating sites will be obtained from the appropriate regulatory committees. Detailed written information will be provided to the family members regarding the study protocol, the scope of research, and the safety of TCD examination. Since all patients are presumed to have an altered state of consciousness, the ethics committees will waive the requirement for consent, as in Italy relatives are not regarded as legal representatives of the patient in the absence of a formal designation.

##

8.1 Approval by ethics committee: *NP 2762

8.2 Informed consent: Written informed consent will be requested from all surviving patients as soon as they regaine their mental competency.

**9 Statistical analysis**

Receiver operating curve (ROC) and the area under the curve (AUC) will be estimated after measurement averaging over time and as well as using time-dependent ROC. Values of ICPi will be dichotomized using a standard reference value of 22 mmHg. Confidence interval for AUC, sensitivity and specificity will be computed using bootstrapping (B = 10000). The sensitivity is expressed as the probability that a patient with high ICPi (>22 mmHg) would also have a high ICPtcd value, and the specificity as the probability that a patient with normal ICPi (≤22 mmHg) would also have a normal ICPtcd value. Best threshold for marker will be computed using Youden criterion [18–21].

**10 References**

*1. Bullock R, et al: Guidelines for the Management of Severe Traumatic Brain Injury. J Neurotrauma , 3 2007, 24(Suppl 1):S1-S106.*

*2. Vukic M, Negovetic L, Kovac D, Ghajar J, Glavic Z, Gopcevic A: The effect of implementation of guidelines for the management of severe head injury on patient treatment and outcome. Acta Neurochir (Wien) 1999,*

*141(11):1203-8.*

*3. Hesdorffer D, Ghajar J, Iacono L: Predictors of compliance with the evidence-based guidelines for traumatic brain injury care: A survey of United States trauma centers. J Trauma 2002, 52:1202-1209.*

*4. Aaslid R, Markwalder TM, Nornes H. Noninvasive transcranial Doppler ultrasound recording of flow velocity in basal cerebral arteries. J Neurosurg. 1982;57:769–774.*

*5. Tsivgoulis G, Alexandrov AV, Sloan MA. Advances in transcranial Doppler ultrasonography. Curr Neurol Neurosci 2009;9:46–54.*

*6. Saqqur M, Zygun D, Demchuk A. Role of transcranial Doppler in neurocritical care. Crit Care Med. 2007;35:S216–S223.*

*7. Alexandrov AV, Sloan MA, Tegeler CH, et al. Practice standards for transcranial Doppler (TCD) ultrasound. Part II. Clinical indications and expected outcomes. J Neuroimaging. 2012; 22:215–224.*

*8. Alexandrov AV, Sloan MA, Wong LK, et al. Practice standards for transcranial Doppler ultrasound: part I—test performance. J Neuroimaging. 2007;17:11–18.*

*9. Klingelhofer J, Conrad B, Benecke R, et al. Intracranial flow patterns at increasing intracranial pressure. Klin Wochenschr. 1987;65:542–545. Kristiansson et al J Neurosurg Anesthesiol _ Volume 25, Number 4, October 2013 382 | www.jnsa.com r 2013 Lippincott Williams & Wilkins*

*10. Klingelhofer J, Conrad B, Benecke R, et al. Evaluation of intracranial pressure from transcranial Doppler studies in cerebral disease. J Neurol. 1988;235:159–162.*

*11. Hanlo PW, Peters RJ, Gooskens RH, et al. Monitoring intracranial dynamics by transcranial Doppler—a new Doppler index: trans systolic time. Ultrasound Med Biol. 1995;21:613–621.*

*12. Aaslid R, Lundar T, Lindegaard K-F, et al. Estimation of cerebral perfusion pressure from arterial blood pressure and transcranial Doppler recordings. In: Miller JD, Teasdale GM, Rowan JO, Galbraith SL, Mendelow AD, eds. Intracranial Pressure VI. Berlin: Springer-Verlag, 1986;229–31.*

*13. Czosnyka M, Matta BF, Smielewski P, et al. Cerebral perfusion pressure in head-injured patients: a noninvasive assessment using transcranial Doppler ultrasonography. J Neurosurg 1998;88:802–8.*

*14. Schmidt EA, Czosnyka M, Matta BF, et al. Non-invasive cerebral perfusion pressure (nCPP): evaluation of the monitoring methodology in head injured patients. Acta Neurochir Suppl 2000;76:451–2.*

*15. Schmidt B, Klingelhofer J, Schwarze JJ, et al. Noninvasive prediction of intracranial pressure curves using transcranial Doppler ultrasonography and blood pressure curves. Stroke 1997;****28****:2465–72.*

*16. Carney N, Totten MA, O’Reilly C, et al. Guidelines for the management of severe traumatic brain injury, Fourth Edition. Neurosurgery. 2016;0:1–10. doi:10.1227/NEU.0000000000001432.*

*17. Stocchetti N, Maas AI. Traumatic intracranial hypertension. N Engl J Med. 2014;371(10):972. doi:10.1056/NEJMc1407775.*

**References Statistics**

*18. Lawrence I-Kuei Lin (1989) A concordance correlation coefficient to evaluate reproducibility. Biometrics, 45 255-268*

*19. Bland JM, Altman DG (1986). "Statistical methods for assessing agreement between two methods of clinical measurement". Lancet* ***327*** *(8476): 307–10.*

*20. Donner A, Rotondi MA. (2010). Sample Size Requirements for Interval Estimation of the Kappa Statistic for Interobserver Agreement Studies with a Binary Outcome and Multiple Raters. International Journal of Biostatistics 6:31*

*21. Buderer NM. Statistical methodology: I. Incorporating the prevalence of disease into the sample size calculation for sensitivity and specificity. Academic emergency medicine : official journal of the Society for Academic Emergency Medicine. 1996 Sep;3(9):895-900.*

*A. Flahault, M. Cadilhac, and G. Thomas (2005). Sample size calculation should be performed for design accuracy in diagnostic test studies. Journal of Clinical Epidemiology, 58(8):859-862.*

*H. Chu and S.R. Cole (2007). Sample size calculation using exact methods in diagnostic test studies. Journal of Clinical Epidemiology, 60(11):1201-1202.*

**11 APPENDIX**

FVd-mca

* CPP*estimated = MABP x ------------- + 14*

FVm-mca

** ICP*estimated = MABP -* CPP*estimated*

*Notes. Grading scores to be used for data gathering*

**MARSHALL score for TBI CTscan**

- DIFFUSE INJURY I: (no visible pathology), ◦no visible intracranial pathology;

• DIFFUSE INJURY II: ◦midline shift of 0 to 5 mm, ◦basal cisterns remain visible, ◦no high or mixed density lesions >25 cm3;

- DIFFUSE INJURY III: (swellig)◦midline shift of 0 to 5 mm ◦basal cisterns compressed or completely effaced ◦no high or mixed density lesions >25 cm3;

• DIFFUSE INJURY IV: (shift)◦midline shift > 5mm, ◦no high or mixed density lesions >25 cm3;

• EVACUATED MASS LESION V: ◦any lesion evacuated surgically;

• NON-EVACUATED MASS LESION VI: ◦high or mixed density lesions >25 cm3, ◦not surgically evacuated

**FISHER score for SAH CTscan (modified)**

- GRADE 0: ◦no subarachnoid haemorrhage (SAH), ◦no intraventricular haemorrhage (IVH), ◦incidence of symptomatic vasospasm: 0% 3;
- GRADE 1: ◦focal or diffuse, thin SAH, ◦no IVH, ◦incidence of symptomatic vasospasm: 24%;
- GRADE 2: ◦thin focal or diffuse SAH, ◦IVH present, ◦incidence of symptomatic vasospasm: 33%;
- GRADE 3: ◦thick focal or diffuse SAH, ◦no IVH, ◦incidence of symptomatic vasospasm: 33%;
- GRADE 4: ◦thick focal or diffuse SAH, ◦IVH present, ◦incidence of symptomatic vasospasm: 40%; Note: thin SAH is < 1 mm thick and thick SAH is >1 mm in depth.

**WFNS for SAH**

- GRADE 1: GCS 15, no motor deficit;
- GRADE 2: GCS 13-14 without deficit;
- GRADE 3: GCS 13-14 with focal neurological deficit;
- GRADE 4: GCS 7-12, with or without deficit;
- GRADE 5: GCS <7 , with or without deficit;

**NIHSS**

1.level of consciousness (1a: 0-3, 1b: 0-2 and 1c: 0-2); 2.best gaze (0-2); 3.visual fields (0-3); 4.facial palsy (0-3); 5.arm motor (0-4); 6.leg motor (0-4); 7.limb ataxia (0-2); 8.sensory (0-2); 9.best language (0-3); 10.dysarthria (0-2); 11.extinction and inattention (0-2);

THESE 11 COMPONENTS ARE THEN SUMMED AND THE SCORE CORRELATES WITH STROKE SEVERITY. •0 = no stroke symptoms; •1-4 = minor stroke; •5-15 = moderate stroke; •16-20 = moderate to severe stroke; •21-42 = severe stroke
