# Supplementary material for: Transcranial Doppler as a screening test to exclude intracranial hypertension in brain-injured patients: the IMPRESSIT-2 prospective multicenter international study
Source: Crit Care. 2022 Apr 15;26:110. doi: 10.1186/s13054-022-03978-2 (PMC9012252; doi:10.1186/s13054-022-03978-2)
Supplement: Supplementary file 4 — Additional file 4. Table S1. Number of measurements of invasive intracranial pressure (ICPi) and trans-cranial Doppler (TCD)-estimated ICP (ICPtcd) at the three time-frames (T1, T2 and T3) in relation to the three ICP different thresholds (> 20 mmHg, > 22 mmHg and > 25 mmHg). [file 13054_2022_3978_MOESM4_ESM.docx]

| **ICP Thresholds** |  | **ICPi - N of measurements (%)** | | | | | **ICPtcd - N of measurements (%)** | | | | | | | | |
| --- | --- | --- | --- | --- | --- | --- | --- | --- | --- | --- | --- | --- | --- | --- | --- |
|  |  | **Time 1** | | **Time 2** | **Time 3** | | **Overall** | | **Time 1** | **Time 2** | **Time 3** | | **Overall** | |  |
| 20 mmHg | **≤20** | 160 (72.1) | 200 (83.3) | | 184 (81.8) | 214 (81.7) | | 141 (63.5) | | 164 (68.3) | | 149 (66.2) | | 172 (65.6) | |
|  | **>20** | 62 (27.9) | 40 (16.7) | | 41 (18.2) | 48 (98.5) | | 81 (36.5) | | 76 (31.7) | | 76 (33.8) | | 90 (98.1) | |
| 22 mmHg | **≤22** | 169 (76.1) | 209 (87.1) | | 193 (85.8) | 232 (88.5) | | 149 (67.1) | | 171 (71.2) | | 162 (72.0) | | 189 (72.1) | |
|  | **>22** | 53 (23.9) | 31 (12.9) | | 32 (14.2) | 30 (11.5) | | 73 (32.9) | | 69 (28.7) | | 63 (28.0) | | 73 (27.9) | |
| 25 mmHg | **≤25** | 190 (85.6) | 219 (91.2) | | 206 (91.6) | 245 (93.5) | | 168 (75.7) | | 195 (81.2) | | 175 (77.8) | | 215 (82.1) | |
|  | **>25** | 32 (14.4) | 21 (8.8) | | 19 (8.4) | 17 (6.5) | | 54 (24.3) | | 45 (18.8) | | 50 (22.2) | | 47 (17.9) | |

**Table S1.** Number of measurements of invasive intracranial pressure (ICPi) and trans-cranial Doppler (TCD)-estimated ICP (ICPtcd) at the three time-frames (T_1_, T_2_ and T_3_) in relation to the three ICP different thresholds (>20 mmHg, >22 mmHg and >25 mmHg).
