# Supplementary material for: Transcranial Doppler as a screening test to exclude intracranial hypertension in brain-injured patients: the IMPRESSIT-2 prospective multicenter international study
Source: Crit Care. 2022 Apr 15;26:110. doi: 10.1186/s13054-022-03978-2 (PMC9012252; doi:10.1186/s13054-022-03978-2)
Supplement: Supplementary file 5 — Additional file 5. Figure S2. Forest plot indicating the areas under a curve (AUC), sensitivity and specificity for each time frame (T1, T2, T3). [file 13054_2022_3978_MOESM5_ESM.docx]

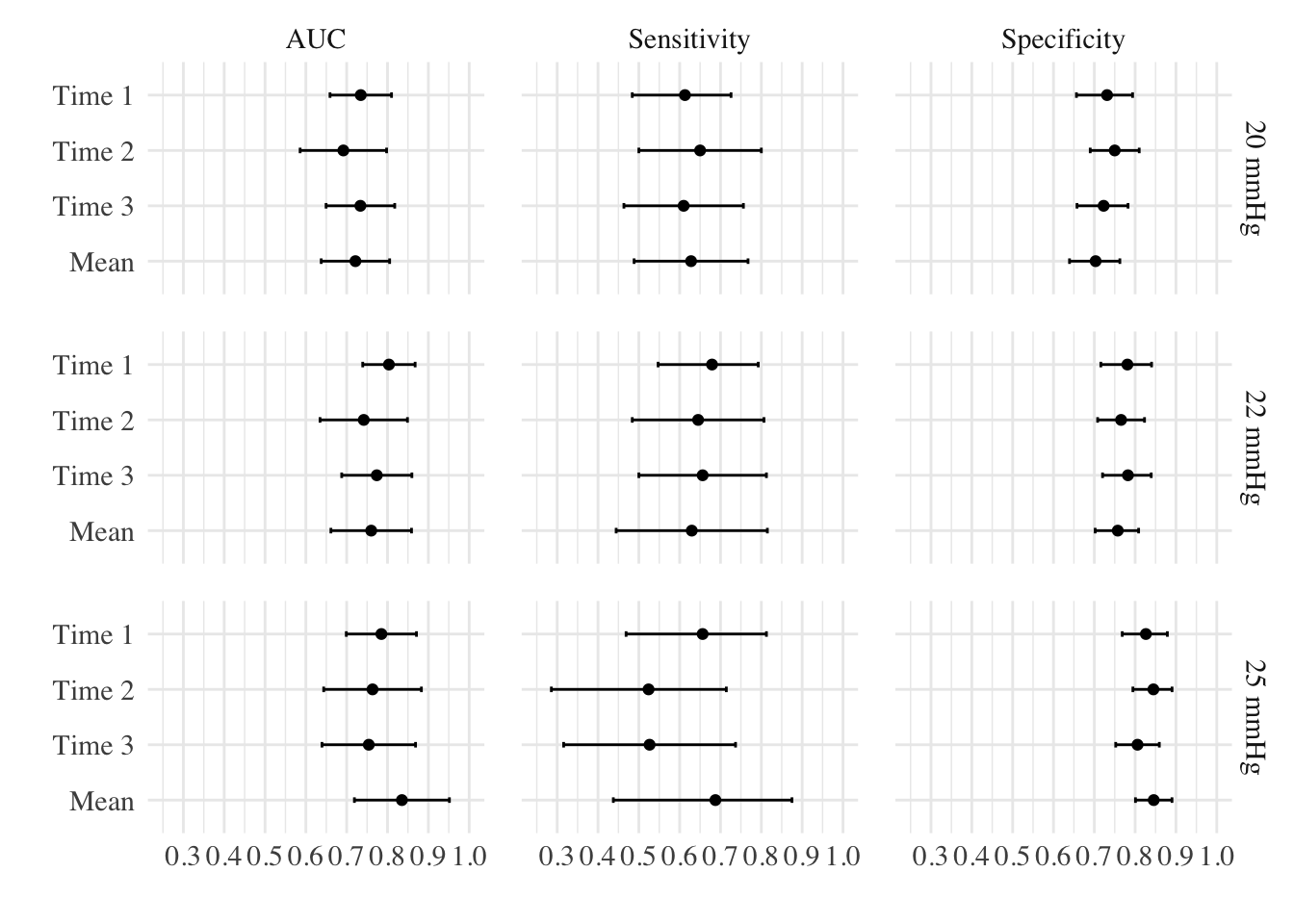


**Figure S2.** Forest plot indicating the areas under a curve (AUC), sensitivity and specificity for each time frame (T_1_, T_2_, T_3_).
