# Supplementary material for: Transcranial Doppler as a screening test to exclude intracranial hypertension in brain-injured patients: the IMPRESSIT-2 prospective multicenter international study
Source: Crit Care. 2022 Apr 15;26:110. doi: 10.1186/s13054-022-03978-2 (PMC9012252; doi:10.1186/s13054-022-03978-2)
Supplement: Supplementary file 6 — Additional file 6. Table S2. Descriptors of diagnostic accuracy of intracranial pressure measured with transcranial doppler (ICPtcd) compared to invasive ICP measurement (ICPi) at three different ICPi thresholds (20, 22 and 25 mmHg) in Traumatic (TBI) vs non-TBI (non-TBI) brain injury. PPV = positive predictive value. NPV = negative predictive value. LR = Likelihood Ratio. AUC = area under the curve. [file 13054_2022_3978_MOESM6_ESM.docx]

| **TBI** | | | | **non-TBI** | | |
| --- | --- | --- | --- | --- | --- | --- |
| **Metric** | **ICP*i =* 20 mmHg** | **ICP*i =* 22 mmHg** | **ICP*i =* 25 mmHg** | **ICP*i =* 20 mmHg** | **ICP*i =* 22 mmHg** | **ICP*i =* 25 mmHg** |
| *Optimal threshold* | 18.5 (12.5-29.5) | 18.5 (14.5-29.5) | 27.5 (14.5-29.5) | 21.5 (12.5-32.5) | 21.5 (11.5-32.5) | 20.5 (20.5-32.5) |
| *sensitivity (%)* | 68.0 (36.0-96.0) | 84.6 (46.2-100.0) | 77.8 (44.4-100.0) | 72.2 (44.4-94.4) | 71.4 (28.6-100.0) | 100.0 (71.4-100.0) |
| *specificity (%)* | 72.3 (35.6-96.0) | 69.9 (45.1-98.2) | 92.3 (53.0-99.1) | 75.4 (39.8-96.6) | 74.6 (30.3-100.0) | 72.1 (61.2-99.2) |
| *ppv (%)* | 37.2 (25.8-70.0) | 24.3 (16.2-75.0) | 42.1 (13.3-80.0) | 31.0 (18.9-66.7) | 25.5 (14.1-100.0) | 16.3 (12.3-85.7) |
| *1-ppv (%)* | 62.8 (30.0-74.2) | 75.7 (25.0-83.8) | 57.9 (20.0-86.7) | 69.0 (33.3-81.1) | 74.5 (0.0-85.9) | 83.7 (14.3-87.7) |
| *npv (%)* | 90.1 (84.8-97.8) | 97.2 (93.4-100.0) | 97.5 (95.5-100.0) | 94.4 (90.8-98.9) | 95.6 (92.4-100.0) | 100.0 (98.4-100.0) |
| *1-npv (%)* | 9.9 (2.2-15.2) | 2.8 (0.0-6.6) | 2.5 (0.0-4.5) | 5.6 (1.1-9.2) | 4.4 (0.0-7.6) | 0.0 (0.0-1.6) |
| *accuracy (%)* | 71.4 (46.8-84.1) | 71.4 (50.0-93.7) | 91.3 (56.3-96.8) | 75.0 (47.0-89.7) | 74.3 (37.5-94.9) | 73.5 (63.2-98.5) |
| *LR+ (sens/1−spec)* | 2.5 (0.6-24.2) | 2.8 (0.8-56.5) | 10.1 (0.9-117.0) | 2.9 (0.7-27.9) | 2.8 (0.4-Inf) | 3.6 (1.8-129.0) |
| *LR- (1−sens/spec)* | 0.4 (0.0-1.8) | 0.2 (0.0-1.2) | 0.2 (0.0-1.0) | 0.4 (0.1-1.4) | 0.4 (0.0-2.4) | 0.0 (0.0-0.5) |
| *AUC (%)* | 71.0 (59.6-82.5) | 78.3 (64.5-92.0) | 79.7 (60.3-99.1) | 71.0 (59.6-82.5) | 78.3 (64.5-92.0) | 88.9 (78.8- |

**Table S2** - Descriptors of diagnostic accuracy of intracranial pressure measured with transcranial doppler (ICP*tcd*) compared to invasive ICP measurement (ICP*i*) at three different ICP*i* thresholds (20, 22 and 25 mmHg) in Traumatic (TBI) vs non-TBI (non-TBI) brain injury.

PPV= positive predictive value. NPV= negative predictive value. LR= Likelihood Ratio. AUC= area under the curve.
