# Supplementary material for: Transcranial Doppler as a screening test to exclude intracranial hypertension in brain-injured patients: the IMPRESSIT-2 prospective multicenter international study
Source: Crit Care. 2022 Apr 15;26:110. doi: 10.1186/s13054-022-03978-2 (PMC9012252; doi:10.1186/s13054-022-03978-2)
Supplement: Supplementary file 7 — Additional file 7. Figure S3. Bland-Altman analysis for each time frame. [file 13054_2022_3978_MOESM7_ESM.docx]

a.
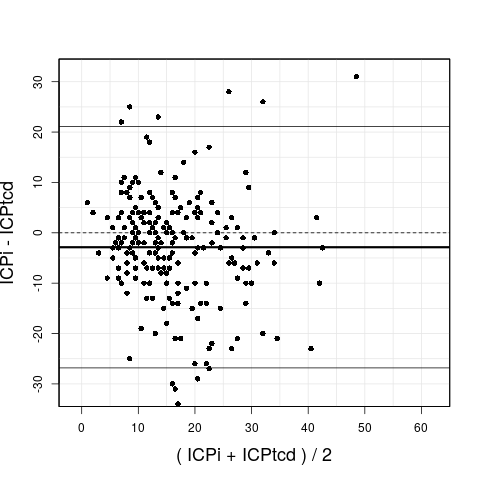
b.
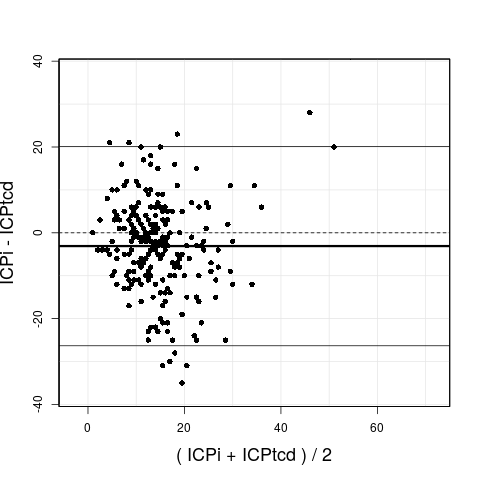


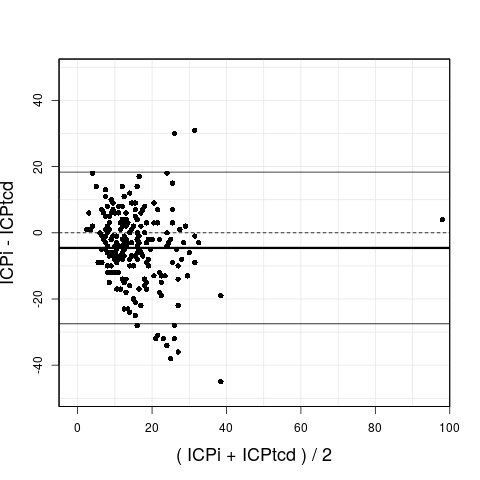

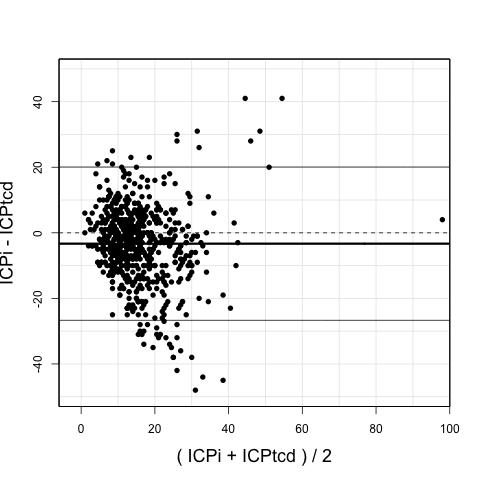


c. d.

**Figure S3.** Bland-Altman analysis for each time frame.

a. = T^1^, b. = T^2^, c.= T^3^, d.= average of all three time frames which yielded a mean bias (ICP*tcd* - ICPi) of -3.3 mmHg with an agreement range comprised between – 26.1 and + 19.5.
