# Supplementary material for: Transcranial Doppler as a screening test to exclude intracranial hypertension in brain-injured patients: the IMPRESSIT-2 prospective multicenter international study
Source: Crit Care. 2022 Apr 15;26:110. doi: 10.1186/s13054-022-03978-2 (PMC9012252; doi:10.1186/s13054-022-03978-2)
Supplement: Supplementary file 8 — Additional file 8. Table S3. Comparison of relevant clinical variables (CPP, TBI vs non-TBI and GCS) in patients with concordant or discordant ICPi and ICPtcd readings at the three ICPi thresholds, (20, 22 and 25 mmHg).CPP showed lower values in the False Negatives and higher values in False Positives compared to Concordant readings. All measurements considered the average value over the the three time-frames (T1–3). [file 13054_2022_3978_MOESM8_ESM.docx]

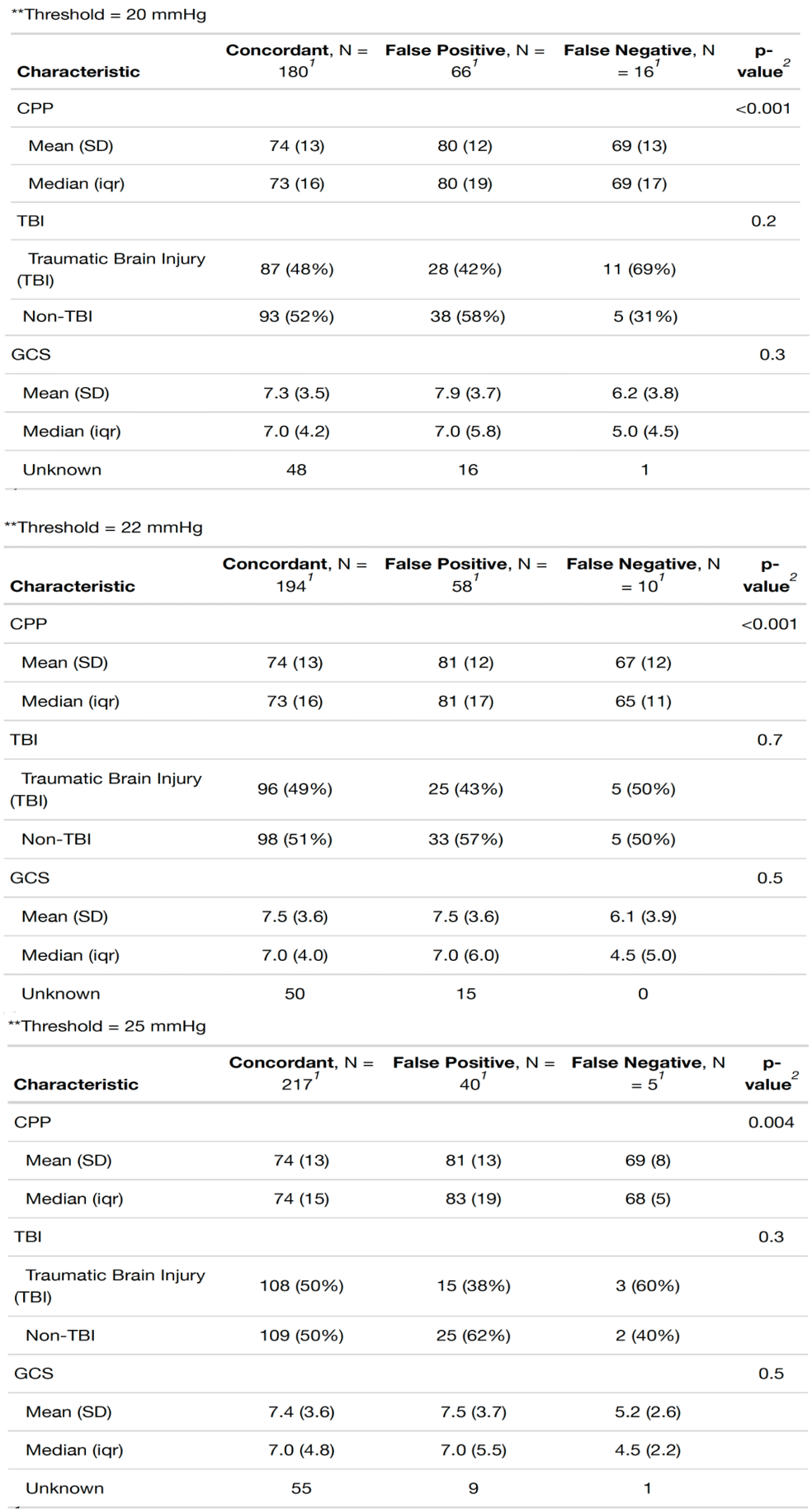


**Table S3.** **Comparison of relevant clinical variables (CPP, TBI vs non-TBI and GCS) in patients with concordant or discordant ICP*i* and ICP*tcd* readings at the three ICP*i* thresholds, (20, 22 and 25mmHg).**

CPP showed lower values in the False Negatives and higher values in False Positives compared to Concordant readings. All measurements considered the average value over the the three time-frames (T_1-3_).
